# Dietary outcomes of community-based CVD preventive interventions: a systematic review and meta-analysis

**DOI:** 10.1017/S1368980023000976

**Published:** 2023-11

**Authors:** Hamid Y Hassen, Binyam G Sisay, Jean-Pierre Van Geertruyden, Delphine Le Goff, Rawlance Ndejjo, Geofrey Musinguzi, Steven Abrams, Hilde Bastiaens

**Affiliations:** 1 Department of Family Medicine and Population Health, Faculty of Medicine and Health Sciences, University of Antwerp, Antwerp 2610, Belgium; 2 Department of Nutrition and Dietetics, School of Public Health, Addis Ababa University, Addis Ababa, Ethiopia; 3 Department of General Practice, University of Western Brittany, Brest, France; 4 Department of Disease Control and Environmental Health, School of Public Health, Makerere University, Kampala, Uganda; 5 Interuniversity Institute for Biostatistics and statistical Bioinformatics, Data Science Institute, Hasselt University, Diepenbeek, Belgium

**Keywords:** Dietary pattern, Community-based intervention, CVD, Systematic review, Meta-analysis

## Abstract

**Objective:**

We aimed to synthesise available evidence on the effects of community-based interventions in improving various dietary outcome measures.

**Design:**

Systematic review and meta-analysis.

**Setting:**

We searched databases including Medline, EMBASE, PSYCINFO, CINAHL and the Cochrane registry for studies reported between January 2000 and June 2022. The methodological quality of the included studies was evaluated using the Cochrane risk of bias tools for each study type. For some of the outcomes, we pooled the effect size using a random-effects meta-analysis.

**Participants:**

A total of fifty-one studies, thirty-three randomised and eighteen non-randomised, involving 100 746 participants were included.

**Results:**

Overall, thirty-seven studies found a statistically significant difference in at least one dietary outcome measure favouring the intervention group, whereas fourteen studies found no statistically significant difference. Our meta-analyses indicated that, compared with controls, interventions were effective in decreasing daily energy intake (MJ/d) (mean difference (MD): –0·25; 95 % CI: –0·37, –0·14), fat % of energy (MD: –1·01; 95 % CI: –1·76, –0·25) and saturated fat % of energy (MD: –1·54; 95 % CI: –2·01, –1·07). Furthermore, the interventions were effective in improving fibre intake (g/d) (MD: 1·08; 95 % CI: 0·39, 1·77). Effective interventions use various strategies including tailored individual lifestyle coaching, health education, health promotion activities, community engagement activities and/or structural changes.

**Conclusion:**

This review shows the potential of improving dietary patterns through community-based CVD preventive interventions. Thus, development and implementation of context-specific preventive interventions could help to minimise dietary risk factors, which in turn decrease morbidity and mortality due to CVD and other non-communicable diseases.

Non-communicable diseases mainly CVD are major causes of adult morbidity and mortality worldwide^(1)^. In 2019 alone, 18·6 million deaths were due to CVD, predominantly IHD and stroke^(1)^. The burden of CVD largely varies across time and regions which could be due to demographic and socio-economic changes, epidemiological transitions, and changes in lifestyle-related factors resulting from globalisation and industrialisation^(2–4)^.

Unhealthy dietary patterns, along with metabolic and anthropometric determinants, are among the most important behavioural risks of CVD^(1)^. In 2019, diet-related risks were among the top five risk factors for mortality^(1)^. Lifestyle modification, particularly targeting dietary risks, is one strategy to prevent cardiovascular events^(5,6)^. Reduction of excess calorie intake, processed food, and increased intake of fruit, vegetables, and wholegrains have been shown to minimise CVD risk^(5,7)^. Likewise, reduction of saturated fat intake or replacement with polyunsaturated fat and increased intake of fibre are among the dietary recommendations for better heart health^(8)^.

Several countries and international organisations have established healthy dietary guidelines to prevent non-communicable disease, including CVD. Nevertheless, passive dissemination of dietary recommendations alone is generally considered ineffective in changing the intended behaviour^(9)^. Multicomponent interventions through active community engagement can improve an individual’s dietary patterns and reduce CVD burden at the population level^(10,11)^. Community-based CVD preventive interventions aimed at improving dietary patterns and physical activity have been implemented using various strategies. However, comprehensive evidence on the impact of such interventions in improving dietary patterns is limited. Few reviews have highlighted the effectiveness of interventions on dietary outcome measures; however, such studies are limited to specific regions, contexts^(12–14)^ or target populations^(15,16)^. In those reviews, details of the intervention components, implementation strategy and their impact on improving specific dietary patterns were not provided. Thus, we systematically reviewed the types and implementation of community-based preventive interventions for CVD and their effectiveness in improving dietary patterns. The evidence from this review is important for practitioners and researchers to design and implement preventive interventions through improvement of dietary patterns.

## Methods

This work is part of a systematic review under the SPICES project – Scaling-up Packages of Interventions for CVD in selected sites in Europe and Sub-Saharan Africa (https://www.uantwerpen.be/en/projects/spices/), which aimed to synthesise available evidence on the effect of community-based interventions (CBI) in improving behavioural risks and CVD knowledge. This paper specifically summarises the evidence on the effects of such interventions on various measures of dietary patterns. The protocol for this review is registered in the PROSPERO international prospective register of systematic reviews (Reg. Number: CRD42019119885), and the result is presented in line with the Preferred Reporting Items for Systematic Review and Meta-Analyses (PRISMA) 2009 guideline^(17)^. The methodological details are available elsewhere^(18)^, and those relevant to this study are briefly summarised here.

### Information sources and search strategy

Initially, MEDLINE, EMBASE, Cochrane Register of Controlled Studies, CINAHL and PSYCINFO were used as the main databases to identify all studies published from 2000 to 2019. Then, the search was updated until June 2022 to include recent results. Other sources, including thesis online, OpenGrey, ProQuest, CHW Central, Google Scholar, ClinicalTrials.gov and the WHO International Clinical Trials Registry, were also searched for more similar articles. After a preliminary keyword search, we developed a systematic search strategy using terms related to population, intervention and outcomes. The details of the search strategy are available elsewhere^(18)^. In addition, more eligible studies were included from reference lists of the included articles.

### Study screening

Studies were eligible to be included in this review if they aimed at prevention of CVD and have dietary patterns as one of the outcomes. Studies were eligible if they were individual/cluster randomised controlled trials or controlled quasi-experimental or interrupted time series studies that tested interventions aimed at primordial or primary prevention of CVD. Moreover, studies were included if they involved adult participants aged 18 years or above; and the interventions were based in community and/or primary healthcare settings. Studies were excluded if participants had diagnosed CVD; interventions included clinical and/or pharmacologic components, with sample size below 150, retention rate below 60 % and a follow-up period shorter than 9 months. Studies that were reported in the English language were considered with no limitation on study location.

**Fig. 1:**
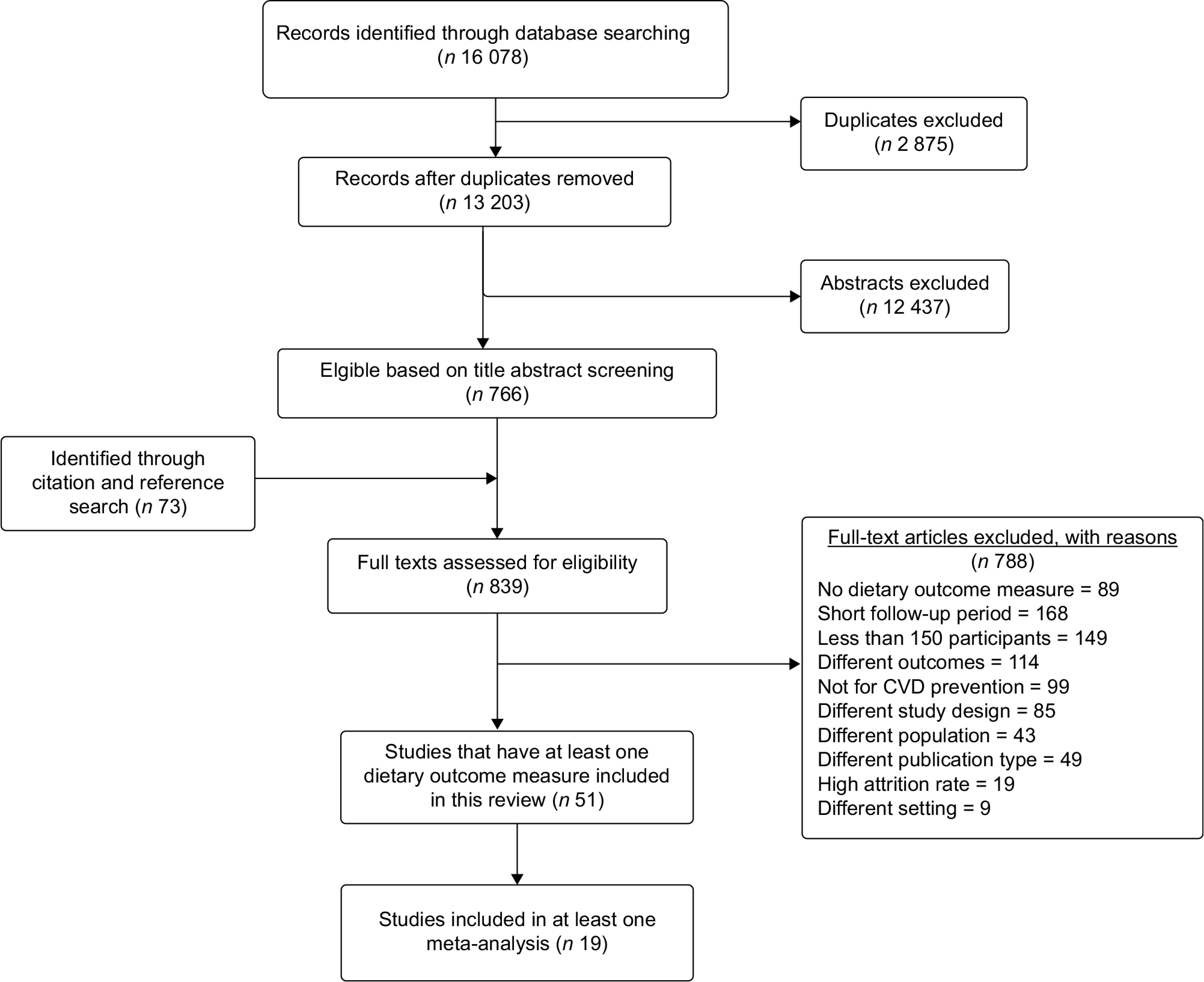
PRISMA flow chart illustrating the article selection process

Endnote files from all databases were checked for duplication, and deduplication was performed using Bramer’s method^(19)^. The deduplicated articles were exported into rayyan.QCRI.org^(20)^ for further deduplication and screening purposes. We performed double screening (HYH and RN/BGS) on all retrieved titles/abstracts using defined criteria. Then, articles included in full-text review were read thoroughly by two independent reviewers (HYH and BGS), and a final decision for inclusion was made. Disagreements between two reviewers were solved through discussion. The article selection process is outlined in the PRISMA flow chart (see Fig. 1).

### Risk of bias assessment and data extraction

For RCT, the revised Cochrane tool for Risk of Bias (RoB2)^(21)^, while for NRC studies the Risk of Bias In Non-randomised Studies – of Interventions (ROBINS-I) tool^(22)^ were used to assess risk of bias of included studies. Double risk of bias assessment (HYH and BGS/RN) was performed independently, and differences were resolved through consensus.

Relevant information was extracted from included articles by two reviewers (HYH and BGS) independently, and disagreements were resolved through consensus. Data on year and country of study, intervention characteristics (description, setting, approach, duration, etc.), study design, participant characteristics, control group, sample size, attrition rate, outcome measures and summary findings were also captured. Furthermore, the outcome measures, summary measures and effect estimates were extracted. Whenever necessary, authors of included studies were contacted for further information. Results that were presented only graphically were extracted using WebPlotDigitizer^(23)^.

### Data analysis

Findings are descriptively presented and discussed by study design, risk of bias, country and income per capita, intervention approach, and outcome measurements. Whenever needed, tables were used to present data comparing country, year of study, intervention duration, context and outcomes.

We used both narrative and quantitative synthesis to summarise evidence in this review. Studies were evaluated for eligibility to be included in the meta-analysis assessing the homogeneity of intervention and outcome measurements. Studies reported several measures of dietary patterns, and we performed a meta-analysis for any measure with at least two studies. As a result, meta-analysis was performed for intake of energy (MJ/d), fat (% of energy), saturated fat (% of energy), fibre (g/d), and fruit and vegetable (servings/d). Findings from studies without sufficient information on the above-mentioned outcome measures or those with other measures of dietary pattern were summarised narratively.

### Meta-analysis

Due to heterogeneity observed in study populations and intervention duration, we expected between study heterogeneity and we performed a random-effects meta-analysis^(24)^ for most of the outcome measures. Mean differences (MD) with 95 % CI were used to summarise continuous outcomes. Whenever needed, standard deviations and/or standard errors for MD were calculated from other reported parameters based on the Cochrane guideline^(25)^. We used the I^2^ statistic to quantify heterogeneity, and we tested the significance thereof using Cochran’s Q statistic^(26)^. We explored the variation in effectiveness across time using subgroup analysis based on follow-up time (9–12 months, 18–24 months, and 36 months and above) and study design for each outcome measure included in the meta-analysis.

We constructed funnel plots to evaluate publication bias graphically, and the significance of symmetry was tested using Egger’s regression test^(27)^. We used the meta-package in the free statistical software package R version 4.0.2 for all the analyses^(28)^. The review results are reported in accordance with the PRISMA 2009 statement^(29)^, and a completed PRISMA checklist is available in the supplementary material (online Supplementary Table S3).

## Results

From all databases, a total of 16 078 titles/abstracts were retrieved (15 885 from initial search and 193 recently updated). Seven hundred and sixty-six articles were retained based on abstract screening, and seventy-three more studies were identified through manual reference searching. Based on the full-text review, fifty-one studies involving 100 746 (56 689 in intervention and 44 057 in control group) reported at least one measure of dietary patterns and were eligible to be included in the narrative synthesis. Of these studies, nineteen were eligible for a meta-analysis with regard to at least one dietary outcome measure. The article screening process is summarised using the PRISMA flow chart (Fig. 1).

### Study characteristics

Detailed characteristics of included studies are available in the supplementary material (online Supplementary Table S1). Of fifty-one studies included in this review, twenty-eight focused on high-income countries, specifically twelve in the USA^(30–41)^, four in the Netherlands^(42–45)^, two each in the UK^(46,47)^, Spain^(48,49)^, and Australia^(50,51)^, and one each in Japan^(52)^, Italy^(53)^, Denmark^(54)^, Germany^(55)^, Sweden^(56)^ and Finland^(57)^. In contrast, twenty-three were in low- and middle-income countries, particularly five in China^(58–62)^, four in India^(63–66)^, three in Iran^(67–69)^, two each in Sri Lanka^(70,71)^ and Kenya^(72,73)^, one each in Bangladesh^(74)^, Nepal^(75)^, Malaysia^(76)^, Pakistan^(77)^, Thailand^(78)^, and Vietnam^(79)^, and one study recruited participants living in China, India and Mexico^(80)^.

Regarding the study design, thirty-three studies were randomised, of which twenty-one and twelve, respectively, were individual- and cluster-randomised. Whereas, eighteen studies were non-randomised controlled studies. Out of thirty-three randomised studies, eight have low, twenty-two some concerns and three high risk of bias based on the Cochrane RoB2 tool. Of eighteen non-randomised studies, two has low, thirteen moderate and three serious risk of bias. The risk of bias summary tables and figures are presented in the supplementary material (online Supplementary Table S4 and Fig. S1).

Several continuous dietary outcome measures were reported including energy intake (MJ/d), Na intake, salt intake, fat (% of energy), saturated fat (% of energy), fibre (g/d), carbohydrate (% of energy or g/d), protein (% of energy or g/d), frequency of sugary beverages, salty diet, fast and/or fried food, fruit and vegetable (servings per d), number of days eat fruit and/or vegetable, healthy eating index, plant-based diet index and diet score. Categorical measures were also reported such as attainment of the required daily fruit and vegetable intake, recommended level of salt, adherence to dietary advice, vegetable procurement, recommended level of sugar, high salt intake, Mediterranean diet, snacks ≥ twice/d, etc. Details of the outcome measurement for individual studies are presented in the supplementary material (online Supplementary Table S1).

### Interventions

Various strategies were employed to deliver the intervention package to target participants and/or populations. Most of them used various health education and awareness creation activities, including seminars, lectures and workshops as the main components of intervention^(30,34,35,37,39,41,46–48,50,52,53,55,56,58–62,65,67–69,72–78,80)^. Furthermore, other strategies were also considered including individual-tailored coaching interventions through face-to-face, mHealth or web-based^(31,33,40,45,46,52,54,56,60,64–66,69,74–76,78,79)^, motivational interviewing^(33,45,48)^, group interactive sessions and/or activities^(30,38,40,42,46,49,51,53,54,63,65,69,71,74,76,78)^, print or electronic materials^(31,39,43,44,55,60,62,69)^, peer support^(32,39,50,63)^, campaigns and mass media^(44,50,66,72,77,79)^, and posters, brochures and pamphlets^(43,44,66,67,69)^. Likewise, health promotion activities through community mobilisation, community networks, structural changes and policy measures^(34,35,47,51,56,59,68,69,80)^ were employed. Details of intervention strategies used by each included studies are available in the supplementary material (online Supplementary Table S2).

Eight studies had an intervention duration ranging from 6 to 9 months^(37,39,41,43,49,55,65,72)^, eighteen studies for 12 months^(30–33,38,45,47,48,51–53,63,70,71,75–78)^, five studies for 14–18 months^(35,40,60,73,74)^, ten studies for 24 months^(34,36,42,46,58,59,62,64,66,80)^, six studies for 36–42 months^(50,57,61,67,69,79)^ and four for 5 years or above^(44,54,56,68)^. The majority of studies followed up participants for outcome measures at 12, 24 and 36 months post-intervention. Most interventions were based in the community-targeting groups of individuals, followed by home-based strategies either face to face or electronically, schools and workplaces or a combination of two or more settings. Trained volunteers, community health workers, peers, healthcare practitioners, nutritionists and other professionals were involved in facilitating the intervention.

Studies employed various dietary outcome measures, including total energy intake (per d), fruit and vegetable servings, fat and/or carbohydrate % of energy, fibre intake, soda/sugary beverage consumption, cholesterol, saturated/unsaturated fat intake, salt intake, Mediterranean diet, healthy eating index, diet score, and frequency of fast food and/or snacks.

### Meta-analysis

**Fig. 2:**
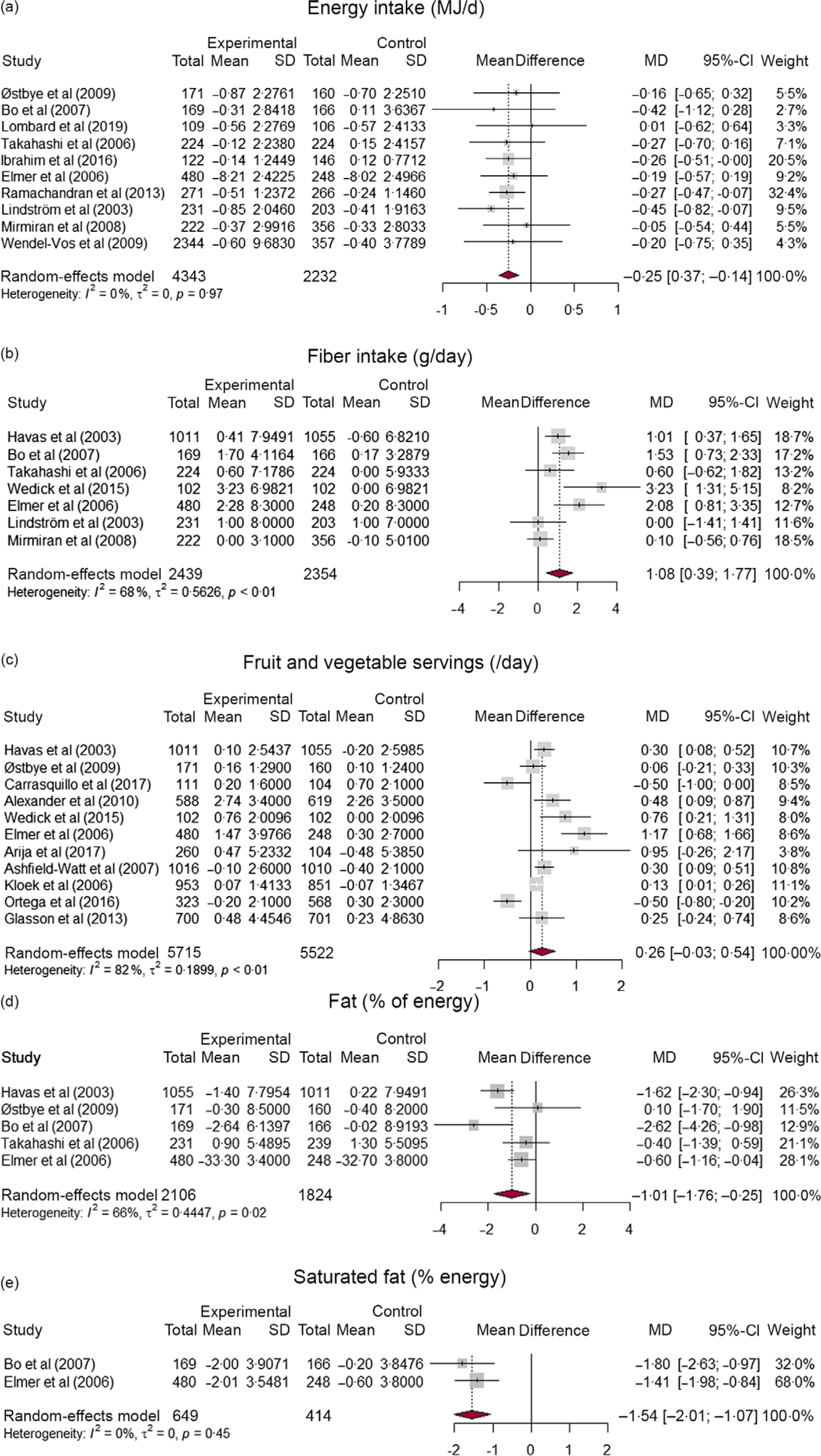
Forest plots indicating the effect of community-based CVD preventive interventions on (a) energy intake, (b) fibre intake, (c) fruit and vegetable servings per d, (d) fat % of energy, and (e) saturated fat % of energy

The pooled effects of CBI with respect to selected dietary outcome measures are summarised in Table 1. In total, nineteen studies were included at least once for one of the five dietary outcome measures that were synthesised. Studies that reported a change in total energy intake (MJ/d), fruit and vegetable intake (servings/d), fibre intake (g/d), fat (% of energy) and saturated fat (% of energy) were considered. Based on ten studies, interventions led to a decrease in daily energy intake compared with controls (MD: –0·25; 95 % CI: –0·37, –0·14; number of studies (n) = 10; I^2^ = 0 %), which is equivalent to 59·8 kilo calories lower intake of energy per d. The pooled results of seven studies showed a 1·1 grams of higher fibre intake per d in the intervention groups compared with controls (MD: 1·08; 95 % CI: 0·39, 1·77; *n* 6; I^2^ = 68 %). A pooled analysis of five studies (all RCT) indicate that the decrease in fat % (MD: –1·01; 95 % CI: –1·76, –0·25; *n* 5; I^2^ = 66 %) and saturated fat % (MD: –1·54; 95 % CI: –2·01, –1·07; *n* 2; I^2^ = 0 %) of daily energy was higher in the intervention group as compared with controls. The increase in fruit and vegetable servings per d was higher in the intervention group compared with control, but the difference was not statistically significant (MD: 0·26; 95 % CI: –0·03, 0·54). Forest plots of all synthesised dietary outcome measures are presented in Fig. 2(a)–(e).

**Table 1:** Pooled effects of community-based interventions on dietary outcome measures

Outcome measure	No. of studies	MD	95 % CI	I^2^	95 % CI
Energy intake (MJ/d)	10	−0·25	–0·37, –0·14***	0	0–62
Fibre intake (g/d)	7	1·08	0·39, 1·77**	68	30–86
Fruit and vegetable (serving/d)	11	0·26	–0·03, 0·54	82	68–89
Fat (% energy)	5	−1·01	–1·76, –0·25**	41	10–87
Saturated fat (% energy)	2	−1·54	–2·01, –1·07***	0	0–0

CI, Confidence interval; MD, mean difference; MJ, mega joule; FU, follow-up; I^2^, Heterogeneity statistic.

** *P* < 0·01;

*** *P* < 0·001.

The subgroup analysis indicated that higher intervention effect in increasing fibre intake at 9–12 months (MD: 1·29; 95 % CI: 0·71, 1·88) and 18–24 months (MD: 2·08; 95 % CI: 0·81, 3·35) of follow-up compared with ≥ 36 months (MD: 0·08; 95 % CI: –0·52, 0·68), with statistically significant subgroup difference (*P* < 0·01). The decrease in fat percent of energy was higher at 9–12 months (MD: –1·16; 95 % CI: –2·20, –0·12) than at 18–24 months (MD: –0·60; 95 % CI: –1·16, –0·04), but the subgroup difference is not statistically significant (*P* = 0·36). No time trend was observed in the remaining outcome measures. Forest plots of subgroup analysis are available in the supplementary material (online Supplementary Fig. S2–S5). Further subgroup analysis by study design showed that RCT showed a larger decrease in energy intake (MD: –0·28; 95 % CI: –0·42, –0·14) than NRC studies (MD: –0·21; 95 % CI: –0·42, 0·00), but the subgroup difference is not statistically significant (*P* = 0·61). The increase in fibre intake was slightly higher for RCT (MD: 1·28; 95 % CI: 0·63, 1·93) than NRC studies (MD: 0·10; 95 % CI: –0·56, 0·76), with significant subgroup difference (*P* = 0·01). Likewise, the increase in fruit and vegetable intake was higher in RCT (MD: 0·41; 95 % CI: –0·00, 0·82) than NRC studies (MD: 0·04; 95 % CI: –0·32, 0·41), with no statistically difference between subgroups (*P* = 0·19) (online Supplementary Fig. S6–S8).

We explored the potential of publication bias using Egger’s test of symmetry and funnel plots. Based on Egger’s test, the null hypothesis of symmetry was not rejected at 5 % significance level for energy intake (*P* = 0·392), fibre intake (*P* = 0·332), fruit and vegetable intake (*P* = 0·485) and fat percentage of energy (*P* = 0·855), indicating that no substantial publication bias was observed. Due to a small number of studies included in the meta-analysis, the statistical power of Egger’s test might not be sufficient to detect considerable bias. However, visual inspection of funnel plots of standard errors against observed effect sizes showed no large deviation from symmetry. Funnel plots of all outcome measures are available in the supplementary material (online Supplementary Fig S2(a)–(e)).

### Narrative synthesis

Besides meta-analyses, a narrative synthesis was also employed to incorporate studies not included therein due to different outcome measures. Overall, out of fifty-one studies, thirty-seven studies (twenty-one from high-income countries and sixteen from low- and middle-income countries) found statistically significant differences in at least one dietary outcome measure favouring the intervention group. Whereas fourteen studies (nine from high-income countries and five from low- and middle-income countries) found no statistically significant difference in various dietary outcome measures across intervention and control groups^(32,33,37,38,42,43,47,49,51,59)^. Of thirty studies that measured fruit and vegetable consumption, ten (33·3 %) found no significant difference across intervention groups. One study^(43)^ found a significant increase in vegetable consumption but not fruit intake. A study by Baumann *et al.* ^(54)^ indicated that the improvement in fruit and vegetable intake in the intervention group compared with the control group was greatest at 5 years of follow-up, but at 10 years the difference across groups was not significant. A study in Sweden^(56)^ found no significant difference across intervention groups in most dietary measures, including percentage of energy from fat, carbohydrates, and protein, intake of fruits, vegetables, wholegrain, fish, sweetened beverages or fried potatoes, and overall diet quality (assessed by Healthy Diet Score). However, men in the intervention county decreased intake of sweets to a greater extent than those in control^(56)^.

Studies that showed a significant improvement in dietary outcomes involved various intervention components, including tailored individual lifestyle coaching and interactive sessions by trained professionals mainly dieticians, health education individually or in group, health promotion activities, community engagement activities and/or structural and system changes such as improving access to healthy food. More specifically, effective interventions consisted of one or more of the following intervention components: individual lifestyle coaching based on risk level and using motivational change tools; counselling by trained professionals besides primary care physicians either in practice or home; customised advice, motivational interview and feedback; and visual demonstrations on food portions. In contrast, interventions through mobile text messages alone, written health pamphlets, brochures and booklets, and postal healthy lifestyle guides were relatively less or not effective. At group level, interventions involving regular interactive group sessions and community lifestyle activities were effective. Furthermore, structural changes such as ensuring healthy foods during organisational meetings/events and increasing availability of affordable fresh fruits and vegetables in corner stores were also effective in improving healthy eating among participants. However, healthy cooking interventions in restaurants and cafeterias were not effective. Further details of intervention strategies and direction of effects for included studies are available in the supplementary material (online Supplementary Table S2).

## Discussion

This review summarises the available evidence on the approach, strategies and effectiveness of community-based CVD preventive interventions in improving healthy dietary patterns, which would contribute to halting the burden of CVD and associated premature mortality. We reviewed fifty-one eligible studies, thirty-three RCT and eighteen NRC studies, exploring the intervention components, duration, outcome measures and their effect on dietary patterns. We also conducted meta-analyses for studies with similar dietary outcome measures. Overall, the findings support that energy intake and fat percentage of energy, particularly saturated, could potentially be reduced through CBI targeting both general and high-risk populations. The mean daily fibre intake was also significantly improved in the intervention group compared with the controls. Intervention strategies involving lifestyle coaching, health education, health promotion activities, community engagement activities, and/or structural and systemic changes demonstrated more pronounced effects. Furthermore, the subgroup analysis showed that relatively higher effects on fibre intake were observed at 12 and 24 months than at 36 months and longer, with significant subgroup differences across time.

Excess energy intake is associated with weight gain, which may increase the risk of CVD incidence and mortality^(81,82)^. By suppressing atherosclerosis and protecting heart cells against ischemic damage, energy restriction is associated with a lower rate of CVD events^(83)^. Thus, decreasing energy intake is one of the required outcomes of preventive interventions for CVD. Most of the studies in this review measured energy intake to evaluate the effectiveness of the intervention, and the majority indicated that CBI are effective in decreasing total daily energy intake, which is also supported by our meta-analysis. On average, participants in the intervention group had 59·8 kcal (250·2 kJ) lower energy intake per d compared with controls. The average recommended daily calorie intake of an adult ranges from 2000 to 2500 kcal^(84)^. Thus, CBI decrease daily energy intake of participants by 2·5 % to 3·0 % as compared with controls, which is a significant percentage towards weight reduction provided that the intervention effect is sustained in the long run. Since calorie restriction favourably affects cardiac function^(85)^, CVD preventive interventions should incorporate strategies to limit an individual’s total calorie intake to the required level that is sufficient for energy balance. Nevertheless, energy restriction interventions require self-monitoring of intake and loss through active weight and food measurements. Training and demonstration of participants on self-monitoring of diet and body weight could be vital components of such interventions.

Healthy dietary guidelines recommend a reduction in dietary saturated fat and replacement with polyunsaturated and monounsaturated fat to lower the risk of CVD^(86)^. Our review and meta-analysis showed that interventions were effective in reducing percent of energy from fat, particularly saturated fat. Overall, interventions led to a 1·1 % decrease in percent of daily energy from fat. Nevertheless, crude assessment of ‘fat percentage of energy’ might not be an appropriate measure of healthy dietary pattern, rather, qualitative identification of specific fat type is more informative. Findings on the association between saturated fat intake and heart disease are inconsistent, which would most probably be due to the variation in comparison groups^(87)^. Replacing saturated fats with polyunsaturated fats is strongly associated with a lower risk of CHD^(88)^. However, replacing saturated fats with refined low-quality carbohydrates results in cardiometabolic disorders, including obesity and diabetes, which increase the CVD risk^(89,90)^. Thus, the superficial use of phrases such as ‘fat intake reduction’ as a dietary intervention might be practically misleading. A few studies included in this review measured percent of energy from saturated fat and the meta-analysis showed that interventions decreased percent of daily energy from saturated fat by 1·5 %. Thus, rolling out such CBI would decrease percent of energy from saturated fat. Interventions should explicitly describe the reduction of saturated fats and their replacement with healthier polyunsaturated fats rather than processed carbohydrates.

Increasing consumption of fibre is also recommended to minimise the risk of a range of diseases, including heart diseases and diabetes^(91–93)^. A few studies included in our review evaluated the effects of interventions on fibre intake. Overall, our meta-analysis showed that interventions were effective in increasing daily fibre intake by approximately 1·1 g than controls. Compared with the recommended daily intake of 25–30 g of fibre, interventions led to a decrease by 3·3–4·0 %. Including fibre intake improvement as a dietary intervention strategy could be helpful for the primary prevention of CVD.

It is evident that fruit and vegetable intake is associated with reduced CVD risk, showing a clear dose–response relationship^(1,94)^. Most of the studies included in our review measured fruit and vegetable intake as one of the outcomes. Our narrative synthesis indicated that most studies found a significant improvement in fruit and vegetable consumption measured in various ways. Our meta-analysis specifically on daily fruit and vegetable servings indicated that there was an increase in the average servings per d by 0·26, but the difference was not statistically significant between intervention and control groups. A previous review also found a similar result, that is, the effectiveness in improving fruit and vegetable servings is minimal^(95)^. A change in fruit and vegetable intake can be hampered by several factors, including the access and affordability of fruits and vegetables. Participants’ socio-economic status and environmental conditions, including access to healthier food, determine the effectiveness of lifestyle interventions^(96)^. However, inaccurate measurement of portion size might also be a reason for the insignificant association.

Overall, effective interventions mostly employed tailored individual lifestyle coaching, stage-matched strategies and interactive sessions by professionals, such as dieticians, health education individually or in groups, community engagement activities, health promotion activities, and/or structural and system changes. One study^(73)^ demonstrated the recommended portions to participants using diagrams of full platter and found significant improvements in all dietary measures in the intervention group compared with controls. Furthermore, interventions that involve multiple components are likely to be more effective than those that use one or two strategies. A review by Crane *et al.* also showed that individual-tailored interventions are the most effective behavioural interventions^(97)^. Thus, tailoring interventions to individual needs and readiness to change involving professionals and practical demonstrations is vital for improving effectiveness.

In general, CBI delivered through various strategies have demonstrated effectiveness in improving various measures of dietary pattern; however, studies have focused on high-income countries. Despite measurement of dietary behaviour being complex, consistent changes were observed following the interventions. Nevertheless, interventions need to emphasise practical demonstrations of dietary intake measurements, including portions of food and energy balance, to observe the intended behavioural change. Our review focused on interventions that measured effectiveness beyond 9 months to depict intermediate- and long-term effects and found significant differences between persons who were subject to CBI and those who were not in most dietary outcome measures. Thus, integrating dietary components along with other lifestyle interventions such as physical activity, cessation of smoking and alcohol consumption could help to reduce the burden of CVD and risk factors at the population level^(18,98)^.

### Methodological considerations

We assessed the risk of bias of studies using tools from the Cochrane Collaboration. However, this quality assessment was hampered by inadequate reporting of each component, particularly reporting of bias from the intended intervention and bias due to missing outcome data. For randomised studies, sequence generation, methods of allocation concealment and blinding were not well described in some of the eligible studies. For non-randomised studies, an inadequate description of study participant selection and insufficient list of confounders and how they were adjusted were among the issues that affected the risk of bias assessment. We recommend the use of standard guidelines to accurately report methodological processes to ensure appropriate interpretation of results and to provide replicable methods for future similar studies.

Furthermore, we considered individual RCT, cluster RCT and NRC studies in the analysis. The unit of randomisation and sampling is different for these study designs, and thus the CI for the effect size might be narrow because clustering would not be taken into account. Nevertheless, we used a Hartung–Knapp–adjusted Sidik–Jonkman method to estimate CI, which is a conservative approach, and the results are less likely to be biased.

### Limitations

By using a systematic approach and two independent reviewers throughout the process, our methodology was strengthened. Nevertheless, interpretation of findings from this review should consider the following limitations. First, restriction of articles to only the English language might have resulted in language bias. Second, owing to the heterogeneity in outcome measurement techniques and inconsistent reporting, we could not perform a meta-analysis for some of the outcomes. Nevertheless, these outcomes were summarised using narrative synthesis. Third, the observed effects of a few outcome measures seems heterogeneous. However, we constructed CI using the Hartung–Knapp-adjusted Sidik–Jonkman method, which resulted in more conservative intervals in case of a small number of studies and large heterogeneity^(99)^.

## Conclusions

This review shows that community-based CVD preventive interventions have the potential of improving dietary patterns and, in turn, CVD risk profiles among adults. Interventions appear to decrease individuals’ daily energy intake, fat and saturated fat percentage of energy, and increase intake of fibre, fruits, and vegetables. A decline in effect size was observed at a longer follow-up, indicating low sustainability after the intervention duration. Intervention components with tailored lifestyle coaching, individual and/or group health education, community-wide health promotion activities, and/or structural and systemic changes such as improving availability of affordable fresh fruits and vegetables in corner stores demonstrated more pronounced effects. Thus, development and implementation of context-specific preventive intervention is beneficial to improve dietary factors, which in turn decrease morbidity and mortality associated with CVD and other non-communicable diseases. Furthermore, favourable intervention effects need to be sustained for longer through linkages with existing primary care centres or community organisations.

## Supplementary material

For supplementary material accompanying this paper visit https://doi.org/10.1017/S1368980023000976. click here to view supplementary material
