# Supplementary material for: Dietary outcomes of community-based CVD preventive interventions: a systematic review and meta-analysis
Source: Public Health Nutr. 2023 May 16;26(11):2480–91. doi: 10.1017/S1368980023000976 (PMC10641629; doi:10.1017/S1368980023000976)
Supplement: Supplementary file 1 [file S1368980023000976sup001.pdf]

Supplementary Materials: Dietary outcomes of community-based cardiovascular disease preventive interventions: a systematic review and meta-analysis

Hamid Yimam Hassen[a]*, Binyam Girma Sisay[c], Jean-Pierre Van Geertruyden[a], Delphine Le Goff[d], Rawlance Ndejjo[e], Geofrey Musinguzi[e], Steven Abrams[a,b¥], and Hilde Bastiaens[a¥]

[a] Department of Family Medicine and Population Health, Faculty of Medicine and Health Sciences, University of Antwerp, Antwerp, 2610, Belgium

[b] Interuniversity Institute for Biostatistics and statistical Bioinformatics, Data Science Institute, Hasselt University, Diepenbeek, 3590, Belgium

[c] Department of Nutrition and Dietetics, School of Public Health, Addis Ababa University, Addis Ababa, Ethiopia

[d] Department of general practice, University of Western Brittany, Brest, France

[e] Department of Disease Control and Environmental Health, School of Public Health, Makerere University, Kampala, Uganda

*Correspondence to:

Hamid Yimam Hassen (Hamid.hassen@uantwerpen.be)

Department of Family Medicine and Population Health, University of Antwerp, Doornstraat 331 Wilrijk, 2610 Belgium

¥ - These authors share senior co-authorship.

Contents

Contents 2
Study characteristics 3
Intervention strategies 12
PRISMA checklist 15
Cochrane risk of bias 18
Subgroup analysis 22
By follow up time 22
Energy intake 22
Fiber intake 23
Fruit and vegetable intake 24
Fat % of energy 24
By study design 25
Energy intake 25
Fiber intake 25
Fruit and vegetable intake 26
Publication bias assessment 27
Search strategy 29

Study characteristics

Table S1. Characteristics of included studies

Author, year, country	Intervention		Participant age (range or mean (SD))	Sample size		Outcome measure(s)	Direction of effect[¥]	Summary of findings
	Duration (months)	Setting		Int.	Cont.			
**Individual randomized controlled studies**								
Alexander et al. (2010), USA	12	Web-based	21–65	Arm 2 = 839; Arm 3 = 838	836	Fruit and Vegetable Servings Per Day	NS	Average servings increased by more than 2 servings across all study arms (P<.001), with the greatest increase (+2.8 servings) among participants of arm 3 (P=.05, compared with control).
Arija et al. (2017), Spain	9	PHC	Mean: 65.2	260	104	Fruit and Vegetable (Servings/day), beverage (g/day), dairy product (g/day), Meat/fish/egg (g/day), salad cereals (g/day), nuts (g/day), Sweetened Cereals (g/day)	NS	The intervention has increased fruit and vegetable consumption however this change is not significant.
Ayala et al. (2015), USA	12	Home-based	Mean: 56.3 (SD: 11.9)	168	168	5+ fruits & vegetables consumed; High fat foods consumed	NS	Intervention participants reported consuming 5 or more fruits and vegetables per day on more days of the week than usual care participants, but this was not statistically significant (P=.09)..
Baumann et al. (2015), Denmark	60	Population based	≥30	11,708	5,264	Intake of vegetables (g/week); Intake of fruit (g/week)	I	Compared to the control group, participants in the intervention group reported a larger decrease in their intake of saturated fat and an increase in vegetable intake through the five years of intervention. However, improvements in the intake of vegetables achieved during the intervention were not maintained in the longer-term.
Bo et al. (2007), Italy	12	Primary care setting	45-64	187	188	Energy intake (MJ/d); Fat (% of energy); Saturated fat (% energy); Polyunsaturated fat (% energy); Carbohydrates (% of energy); Protein (% of energy); Fiber (g/day)	I	The intervention significantly reduced total/saturated fat intake and increased polyunsaturated fat and fiber intake. No significant change was reported in the controls.

Carrasquillo et al. (2017), USA	12	Community based	18-65	150	150	Fruit and Vegetable Servings Per Day	NS	The intervention doesn't lead to improved intake of fruits and vegetable.
Chao et al. (2012),China	18	Primary care setting	≥60	1,163	1,198	Diet (0–18)	I	Compared with the control group, the management group demonstrated improvement on diet score.
Davies et al. (2016),UK	36	GP practices	25-75	447	433	Fiber intake;<br>Fat intake;<br>Unsaturated fat intake	I	Fat intake was not significantly reduced in the intervention group compared to the standard-care group, but statistically significant increases in unsaturated fat intake were reported.
Elmer et al. (2006), USA	18	Community -based and online	≥25	Established=268; Established + DASH=269	273	Fruit and Vegetable (servings/d; dairy intake (servings/d), fat (% of energy); calorie intake (MJ/d);<br>fiber (g/d), total fat (% of calorie), cholesterol intake (mg/d)	I	Compared with advice only, both behavioral interventions statistically significantly reduced, fat intake. The established plus DASH intervention also statistically significantly increased fruit and vegetable intake.
Havas et al. (2003),USA	6	Home and community based	≥18	1011	1055	Fat (% of energy); fruit and vegetable servings per day;<br>fiber (g/day)	I	There is a significant improvement in dietary outcome measures indicating multiple dietary improvements can be achieved in a low-income population with an effective, multi-faceted intervention program.
Laska et al. (2016), USA	24	Home based	18-35	224	217	Fast food (times/week);<br>Sugary beverages (times/day)	I	The intervention resulted in decreases in fast food consumption, compared to the control condition
Lindström et al. (2003), Finland	36	Primary care setting	40-64	256	250	Energy intake (kcal/d); carbohydrate (%), Fat (g/day); saturated fat (E%), Monounsaturated fat (E%), polyunsaturated fat (E%), Fiber (g/day)	I	The intensive lifestyle intervention produced long-term beneficial changes in diet.
Lu et al. (2015), China	24	Community -based	40–75	RL =120<br>IW= 120	SL= 120	Appropriate salt intake	I	Improvements in appropriate salt intake were progressively greater from self-learning to regular lecture to interactive education workshop.
Okube et al. (2022), Kenya	15	Community -based	18–64	156	138	Dietary intake patterns included frequency of consumption of processed/fast foods, daily servings of fruits and vegetables, legumes, nuts, amount of sugars, and salts intake.	I	Daily consumption improved significantly ($p < 0.001$) for fruits (IG = 44.2% vs CG = 20.3%) and vegetables (IG = 70.5% vs CG = 49.3%) in the intervention compared to the control group by the end line.

								The recommended amount of sugar (IG = 64.7% vs CG = 45.7%) and salt (IG = 69.6% vs CG = 50.0%) significantly (p = 0.001) improved in the IG as compared to the CG by the end-line. The proportion of frequent consumers of legumes (IG = 48.1% vs CG=23.2%) and nuts (IG=31.4% vs CG=15.9%) improved significantly (p < 0.001) in the IG compared to the CG by the end-line.
Østbye et al. (2009), USA	9	Community based	≥18	225	225	Total calories (MJ/d); fat (% of energy); soda/day; sweetened beverages (times/day); fast food (times/week); fries/chips /day; fruit and vegetable servings /day	NS	There were no significant differences among the arms in dietary measures.
Ramachandran et al. (2013), India	24	Home-based	35-55	271	266	Dietary energy intake (kcal); Adherence to dietary advice	I	Total dietary energy intake was lower in the intervention group than in the control group. Moreover, at the end of follow-up, a greater proportion of participants in the intervention group were adherent to diet than in the standard-care group
Takahashi et al. (2006), Japan	12	Population based	40-69	274	276	Energy (kJ); fruit and vegetable intake, Protein (% of energy); Carbohydrates (% of energy); Fat (% of energy); Potassium/Sodium ratio; Fiber (g/day); Soluble dietary fiber; Insoluble dietary fiber; Sodium (mg/day); Potassium (mg/day); Carotene (g/day); Alpha-carotene (g/day); Beta-carotene (g/day); Vitamin C (mg/day);	I	At year 1, intake of fruit and vegetables and of dietary carotene and vitamin C increased significantly more in the intervention group (P < 0.05). Sodium intake in the intervention group decreased by 15 mmol/day (95% CI: -26, -4), but increased by 11 mmol/day (-0, +22) in the control group. This difference in change between the two groups was statistically significant (P=0.002).
van Keulen et al. (2021), Netherlands	12	Home-based	45-70	TPC-405 TMI-407 Combined- 408	409	Fruit intake, Vegetable intake;	I	For fruit consumption, participants in the TPC group were more likely to adhere to the fruit consumption guideline than those in control group, and more participants in the TPC group met this guideline than participants in the combined group. Participants in the TMI group appeared more likely to meet this guideline than those in control group (borderline significance). The following ranking

								seemed to apply: TPC ≥ TMI ≥ combined ≥ control. Regarding vegetable consumption, more participants in the TPC group adhered to the vegetable consumption guideline than those in the combined or control group, with the following ranking: TPC ≥ TMI = combined ≥ control.
Wedick et al. (2015), USA	12	Population based	21-70	102	102	Alternate healthy eating index (0-80); energy intake (kc/day); fat (% of energy); saturated fat (% of energy); carbohydrate (% energy); protein (% of energy); dietary cholesterol (mg/day); dietary fiber (g/day); fruit and vegetables (servings/day); whole grains (servings/day)	I	Adjusting for baseline value of the dietary variable, treatment group, age, income, employment, and social support for healthy eating. All post-intervention changes were statistically significant ($p<0.01$).
Woodruff et al. (2019), USA	12	Home based	35-65	172	177	Healthy eating index	I	Intervention participants reported greater improvements in HEI-2010 total score relative to control participants at 6 months (+3.41 ±13.43 intervention group vs +2.02 ± 12.26 control group) and 12 months of follow-up (+1.73 ± 13.44 intervention group vs +.89 ± 12.66 control group). These improvements were significantly different in growing curve models (P = .009)..
Zhang et al. (2018), China	24	Primary care setting	≥60	337	334	High diet score	I	The intervention has improved the diet score of participants better than controls.
**Cluster randomized studies**								
Bóveda-Fontán et al. (2015),	12	Primary care	40-75	107	120	Mediterranean diet	I	A positive change has been observed in adherence to Mediterranean diet.
Chandraratne et al. (2019), Sri Lanka	12	community based	15-29	303	288	Vegetables, ≥ two servings/day; Fruits, ≥ one serving/day; Snacks, ≥ twice/day; Sugar-sweetened beverages, ≥ once/day	I	The intervention group had a higher probability of consuming at least one serving/day of fruits and a lower probability of consuming snacks twice/day or more than the control group.
Daivadanam et al. (2018), India	9	Community -based	25-45	239	239	Intake of fruit and vegetable, vegetable procurement, consumption of salt, sugar, oil	I	Significant increase in fruit intake in the intervention arm (12.5%) and control (6.6%) but no difference between the groups. Significant

								increase in vegetable intake in intervention (13.99%) and control arms (13.66%) but no difference between the groups. Significant increase in vegetable procurement by 19% in the intervention arm compared to the control arm (p = 0.008). Monthly household consumption of salt, sugar and oil was greatly reduced in the intervention arm (p < 0.001)
Dirige et al. (2013), USA	18	Community based	≥18	255	273	Fruit and vegetables stage (1-5); Dietary fat stage (1-5)	I	Participants showed significant increases in adoption of a low-fat diet, and stage of change for fruit and vegetables, dietary fat intake.
Fottrell et al. (2019), Bangladesh	14	Community -based	≥30	PLA – 3798, mHealth - 3812	3892	Mean number of portions of fruit and/or vegetables consumed per day¶	NS	No significant difference in mean number of portions of fruits and vegetables consumed per day in both PLA (0.29 (0.10–0.69; p = 0.143) and m health (−0.19 (−0.53 to 0.15; p = 0.274) groups compared to control
Gunawardena et al. (2016), Sri Lanka	12	School and home-Based	≥18	152	156	Consumption of fruits and vegetables, whole grain, fried food, sugar sweetened beverages	NS	No significant difference in consumption of fruits and vegetables, whole grain, fried food, sugar sweetened beverages between the two groups
Joshi et al. (2012), India	24	Combined (PHC, community)	≥30	592	543	Number of days eat fruit, green leafy vegetables, oily food, salty food	I	Mean number of days eat oily food was significantly lower in the intervention villages than those in controls (p = 0.01). No significant difference in mean no. of days eat fruit, green leafy vegetables and salt consumption
Landry et al. (2017), USA	6	Community -based	≥18	MMA= 167	SMA= 152	Healthy eating index - 2005	I	Significant treatment effects were present for two components—total and whole fruit; scores were higher in the multiple- message approach arm as compared to the single-message approach arm across time points.
Lombard et al. (2010), Australia	12	School based	25-51	127	123	Energy intake (MJ/d); Total fat, g/day; Saturated fat (g/d)	NS	The intervention and control group reported significant reduction in energy intake saturated fat and total fat intake. However, no significant difference between intervention and control groups.
Neupane et al. (2018), Nepal	12	Home-based	25-65	939	699	Low fruit and vegetable intake, high salt intake,	NS	No significant differences between the intervention and control groups in proportions of people who consumed ≥5 g of salt each day (0·80, 0·56–1·14) and ate less than five servings of fruit

								and vegetables each day (OR = 1·09, 95% CI: 0 38–3·13).
Thankappan et al. (2018), India	12	Community based	30-60	507	500	5 and above fruits & vegetables consumed	I	Compared with the control group, intervention participants had a greater increase in fruit and vegetable intake (>5 servings/day) (RR=1.83; 95%CI: 1.17, 2.84).
Yokokawa et al. (2020), Thailand	12	PHC	≥35	393	402	Daily salt intake	I	As for estimated daily salt intake, although the change was significantly larger in the intervention group compared to the control group at both follow-ups, the difference became smaller at 12 months compared to 6 months (-0.86 vs -0.22 g/day, P < .01, 95% CI = 0.29-0.98 and -0.93 vs -0.55 g/day, P = .02, 95% CI = 0.06-0.70 at 6 and 12 months, respectively).
**Non-randomized controlled studies**								
Anthony et al. (2015), China, India, and Mexico	24	Community and PHC	18-64	5,442	6,694	≥5 portions fruit and vegetables/day; Added salt at the table	I	The intervention has resulted an increased consumption of fruit and vegetable intake, and reduction in salt intake.
Ashfield-Watt et al. (2007),UK	12	Community -based	Adults	798	268	Total fruit and vegetables (portion)	NS	Total fruit and vegetable intakes decreased significantly over one year in the control group (-0.4 portions per day, P < 0.01), but there was no significant change in total fruit and vegetable intakes in the intervention group
Azizi et al. (2013), Iran	42	Community -based	20-74	2961	3909	Energy intake and macronutrient consumption	NS	No significant difference in energy intake and macronutrient consumption between two groups at baseline and after intervention.
Glasson et al. (2013), Australia	36	Community based	Adults	695	708	Fruit and vegetable servings per day	I	Exposure to the program resulted in a net increase of 0.5 servings of fruit and vegetables daily for those who recalled the program compared with those who did not.
Huang et al. (2011), China	36	community based	≥35	826	806	Salty diet, fat intake, pickled food	I	After 3 years of follow-up, there were significant dietary modifications in group I, such as changes in salt and fat intake. No significant difference in pickled food intake, but there was a significant reduction within group I and an increase within group C.

Ibrahim et al. (2016), Malaysia	12	Community -based	18-65	122	146	Energy (Kcal), Percent who fulfilled the recommended level.	I	Total energy intake decreased significantly in the intervention group than controls (P=0.004). Participants in the intervention group had a higher percentage of participants (13.9%) who met the dietary aims (to reduce 20 ± 25 kcal/kg energy intake) as compared to usual care (9.6%), but the difference was not statistically significant (p=0.268).
Kloek et al. (2006), Netherlands	24	Community based	18-65	958	856	Vegetable servings per day (portion); Fruit and vegetable servings per day	NS	The intervention demonstrated no evidence for an impact on vegetable consumption and weak evidence for a small impact on (intermediate) outcomes of fruit consumption.
Koeder et al. (2022), Germany	6	Community -based	≥18	112	75	Plant-based diet index (PDI), healthful PDI (hPDI) and unhealthful PDI (uPDI)	I	Compared to control, in the intervention group, the 1-year trajectories of PDI and hPDI were higher by 2.7 (95% CI 1.7, 3.6) food portions/day and 3.9 (95% CI 2.7, 5.0) food portions/day, respectively, while the 1 year trajectory of uPDI showed a decrease of -2.7 (95% CI -3.7, -1.7) food portions/day (between-group differences: P < 0.001; adjusted for baseline).
Luten et al. (2016), Netherlands	9	Community and environmen t	≥55	430	213	Fruit servings per day; Vegetable servings per day	NS	No significant changes were found for fruit consumption in the intervention and control groups. However, vegetable consumption has increased significantly within the intervention group. However, the difference across groups is not significant.
Lv et al. (2014),China	24	Community and PHC	18-64	1016	1000	Fruits and vegetables consumption	NS	FV consumption increased significantly in the intervention areas (mean score from 24.8 to 26.0, p=0.036) and the comparison area (mean score from 24.3 to 26.7, p<0.001). However, no significant difference across intervention groups.
Mirmiran et al. (2008), Iran	36	multi settings	18-74	222	356	Energy intake (MJ/d), carbohydrates (g/day), fat (g/day), fiber (g/day), cholesterol (g/day), protein %, carbohydrate %, fat %	I	The mean carbohydrate and fat intakes decreased in both cases and controls, significant in the cases. After adjustment for age, sex, and baseline variables, we found a significant decrease only for dietary total cholesterol (p<0.05).

Nguyen et al. (2012), Vietnam	36	Community based	≥25	2,352	2,298	Salty diet	I	A significant reduction in salty diet for both sexes after 3 years in the intervention community.
Nishtar et al. (2007), Pakistan	12	Community based	18–65	288	304	Consumption of fruits and vegetables (per day), type of oil,	I	Significant differences in consumption of two or more servings of vegetables (per day) between the intervention and control group at the end of the intervention (0.020). No significant differences observed regarding consumption of five or more servings of fruits and vegetables, consumption of two or more fruit servings and type of oil/fat/ghee used for cooking
Ortega et al. (2016), USA	24	Community based	Adult	313	482	Dollars spent on fruits and vegetables per week, Percent of dollars spent on fruits and vegetables, fruits and vegetables servings per day, perceived healthy food accessibility and perceptions of corner stores	I	Improvements were found in perceived healthy food accessibility and perceptions of corner stores. However, no changes were found, however, in store patronage, purchasing, or consumption of fruits and vegetables
Sarrafzadegan et al. (2009), Iran	60	community based	≥19	1,536	1,536	Dietary score, healthy diet score, fruit and vegetable consumption, unsaturated fat, salt intake	I	A significant increasing trend in mean dietary scores in both intervention areas (P for trend < 0.05), but no significant change in the control area (P for trend = 0.41). A similar pattern was seen in the percentage of individuals who ate a healthy diet.<br>After 5 years of interventions, members of the high risk population in the interventional area have improved their nutritional habits with respect to fruit and vegetable consumption more than the reference area (24% increasing vs. 5% increasing, p<0.05). They have used more unsaturated fat and less salt in their diet regimens (p<0.05)
Törmä et al. (2021), Sweden	30 years	Community -based and PHC	25–74	2555	2845	Healthy diet score; servings/d of healthy food items including whole grain, fish, fruits, and vegetables; serving/d of unhealthy food items: red/processed meat, sweets, sweetened beverages, fried potatoes	NS, I	No differences in temporal trend for estimated percentage of energy intake from total carbohydrates, total fat, total protein and alcohol were observed between the counties (P ≥ 0.33). There were no between-county difference in temporal trends for overall diet quality (assessed by the Healthy Diet Score; P = 0.36). Nor were there any between-county differences for the

								intake of whole grain products, fruits, vegetables, fish, sweetened beverages or fried potatoes ($P \geq 0.09$). Consumption of meat ($P = 0.05$) increased to a greater extent in control county from 2009 and onwards, mainly in men (sex-specific analyses, $P = 0.04$). Men in intervention county decreased their intake of sweets to a greater extent than men in control county ($P < 0.01$).
Van de Vijver et al. (2016), Kenya	6	Community based	≥35	1,531	1,233	Insufficient fruit and vegetable consumption	I	Insufficient intake of fruits and vegetables increased significantly at population level both in intervention (OR 1.30, 95% CI 1.08 to 1.56, p=0.006) and control (OR 1.42, 95% CI 1.15 to 1.76, p=0.001) settings.
Wendel-Vos et al. (2009), Netherlands	60	community based	Mean: 50.6 (SD: 9.8)	2,356	758	Energy intake (MJ/d), Fat intake (g/d), Saturated fat (g/d), Polyunsaturated fat (g/d), Mono-unsaturated fat (g/d)	I	The community intervention succeeded in preventing age- and time related unfavorable changes in energy intake, and fat consumption.

MMA: Multiple Message Approach; SMA: Single Message Arm; SL: Self-learning; IW: Interactive workshop; RL: Regular lecture; TPC: computer-tailored print communication; TMI: telephone motivational interviewing; PLA – participatory learning and action; mHealth – mobile health

¥ - I – Intervention; NS – no significant difference; C - control

Intervention strategies

Table S2. Summary of intervention strategies and direction of effect for included studies

Study	Intervention strategies	Direction of effect
**Individual randomized controlled studies**		
Alexander et al. (2010)	Web-based tailored intervention with/out motivational interviewing, short video and audio files	NS
Arija et al. (2017)	Supervised group walking sessions and socio-cultural activities once a month	NS
Ayala et al. (2015)	Peer support: assistance with diabetes management	NS
Baumann et al. (2015)	Tailored individual and/or group-based lifestyle counseling	I
Bo et al. (2007)	Family physician advice, detailed verbal and written recommendations, group sessions	I
Carrasquillo et al. (2017)	Home visits, telephone calls, and group-level activities	NS
Chao et al. (2012)	Health record establishment; Health evaluation; and Health management, tailored advice, education/skills training, telephone consultation, lectures on health, and distribution of health promoting materials	I
Davies et al. (2016)	Tailored and structured education program, group sessions telephone call	I
Elmer et al. (2006)	Counseling, group and individual sessions, self-monitoring	I
Havas et al. (2003)	Interactive sessions using peer educators, written materials	I
Laska et al. (2016)	Academic course, social network and support website	I
Lindström et al. (2003)	Dietary counseling by nutritionist, tailored PA training	I
Lu CH et al. (2015)	Health education booklets, text message, reading materials, regular lecture, interactive education workshop	I
Okube et al. (2022)	Demonstration using a diagram of full platter to depict the recommended portions of carbohydrate, proteins and vegetables/fruits for consumption critical in prevention of CVDs	I
Østbye et al. (2009)	Lecture sessions, telephone counseling sessions	NS
Ramachandran et al. (2013)	Mobile phone messages	I
Takahashi et al. (2006)	Tailored dietary education	I
van Keulen et al. (2021)	Customized stage-matched advice; motivational interviewing; computer algorithm with feedback messages; sending letters	I

Wedick et al. (2015)	Individual and group sessions, Raising awareness and motivating towards healthy lifestyle, diet manual	I
Woodruff et al. (2019)	Coaching, printed materials	I
Zhang et al. (2018)	health education both at individual and community level, family management, community management	I
**Cluster randomized studies**		
Bóveda-Fontán et al. (2015)	Training, motivational interview	I
Chandraratne et al. (2019)	Health promotion activities using youth clubs	I
Daivadanam et al. (2018)	Counselling accompanied by home-visit; phone call, general awareness sessions; stage-matched strategies	I
Dirige et al. (2013)	Health promotion using health education, behavior change skills development, and organizational policy change	I
Fottrell et al. (2019)	Monthly group meeting led by lay facilitator; awareness raising, exercising in groups; income generation and kitchen gardening; training of informal healthcare workers; mHealth intervention	NS
Gunawardena et al. (2016)	Trained health promotion facilitators delivered the intervention in the form of discussion with selected students; Students encourage their family members	NS
Joshi et al. (2012)	Posters, street theater, rallies, and community presentations designed to convey messages about stopping tobacco use, heart-healthy eating, and physical activity	I
Landry et al. (2017)	Nutrition education program, monthly group sessions	I
Lombard et al. (2010)	Group sessions, goal setting, self-monitoring, social support, mobile text message	NS
Neupane et al. (2018)	Training of female community health volunteers (FCHVs); FCHVs visited selected households three times a year (every 4 months) to provide health promotion counselling and to measure blood pressure;	NS
Thankappan et al. (2018)	Peer-support program; group sessions, various community activities	I
Yokokawa et al. (2020)	Health education intervention comprising visualization of the salt content in their typical home-prepared soup and their estimated daily salt intake, and a small group health education class. Dieticians performed the small group education classes at 1 and 3 months after enrollment.	I
**Non-randomized controlled studies**		
Anthony et al. (2015)	Health education, structural change, and community mobilization	I
Ashfield-Watt et al. (2007)	Community networks, community activities, improving awareness, attitude and access	NS
Azizi et al. (2013)	Nutrition education classes (face to face), well-being message - written health newsletter; Pamphlets, brochures and booklets written on smoking, nutrition, physical activity and coping with stress were	NS

	distributed 2-4 times a year to all families; Lectures during religious meetings; face to face interviews; group meetings; advisory clinics; school-based program	
Glasson et al. (2013)	Health education by peer educators, social marketing, school campaign, cooking demonstrations, using volunteers,	I
Huang et al. (2011)	Health education, lifestyle guidance, training local healthcare staff,	I
Ibrahim et al. (2016)	Community-based lifestyle intervention (Co-HELP) - twelve group-based sessions and two individual counselling	I
Kloek et al. (2006)	Nutrition projects in schools, information on healthy nutrition and lifestyle for adults, group health promotion activities, monthly mailed newsletter	NS
Koeder et al. (2022)	Workshops, seminars on lifestyle change predominantly plant-based diet (strongest emphasis), physical activity, stress management and social health; healthy lifestyle handbook, a recipe booklet and a laminated information sheet with an overview of the lifestyle recommendations	I
Luten et al. (2016)	Posters, radio spots and interviews, Advertorials and press reports, newsletters, flyers, printed 'good health' guide and website, environmental approaches	NS
Lv et al. (2014)	Community mobilization, structural change, health education and social marketing	NS
Mirmiran et al. (2008)	Face to face health education at school, health centers, public places, change in school foods, lecture and discussion sessions, Pamphlets and posters	I
Nguyen et al. (2012)	Individual advice, periodic lifestyle promotion campaigns via broadcasting, leaflets or meetings	I
Nishtar et al. (2007)	Community health education, mass media interventions, training of health professionals, health education through Lady health workers	I
Ortega et al. (2016)	Community-engaged, multi-level corner store intervention	I
Sarrafzadegan et al. (2009)	Public education through the mass media, inter-sectoral cooperation and collaboration, community participation, education and involvement of health professionals, marketing and organizational development, legislation and policy development or enforcement, and research and evaluation	I
Törmä et al. (2021)	Individual health assessment and counseling; public awareness on CVD and risks; food labeling system	NS, I
Van de Vijver et al. (2016)	Awareness campaigns, household visits for screening, and referral and treatment of people with hypertension	I
Wendel-Vos et al. (2009)	Printed materials, video guided activities, campaigns, leaflets, posters, targeting both high risk and population based	I

¥ - I – Intervention; NS – no significant difference; C - control

PRISMA checklist

Table S3: PRISMA checklist

Section/topic	#	Checklist item	Reported on page #
**TITLE**			
Title	1	Identify the report as a systematic review, meta-analysis, or both.	1
**ABSTRACT**			
Structured summary	2	Provide a structured summary including, as applicable: background; objectives; data sources; study eligibility criteria, participants, and interventions; study appraisal and synthesis methods; results; limitations; conclusions and implications of key findings; systematic review registration number.	2
**INTRODUCTION**			
Rationale	3	Describe the rationale for the review in the context of what is already known.	3
Objectives	4	Provide an explicit statement of questions being addressed with reference to participants, interventions, comparisons, outcomes, and study design (PICOS).	3
**METHODS**			
Protocol and registration	5	Indicate if a review protocol exists, if and where it can be accessed (e.g., Web address), and, if available, provide registration information including registration number.	4
Eligibility criteria	6	Specify study characteristics (e.g., PICOS, length of follow-up) and report characteristics (e.g., years considered, language, publication status) used as criteria for eligibility, giving rationale.	4, 5
Information sources	7	Describe all information sources (e.g., databases with dates of coverage, contact with study authors to identify additional studies) in the search and date last searched.	4
Search	8	Present full electronic search strategy for at least one database, including any limits used, such that it could be repeated.	4 and supplement Box1
Study selection	9	State the process for selecting studies (i.e., screening, eligibility, included in systematic review, and, if applicable, included in the meta-analysis).	4, 5
Data collection process	10	Describe method of data extraction from reports (e.g., piloted forms, independently, in duplicate) and any processes for obtaining and confirming data from investigators.	5

Data items	11	List and define all variables for which data were sought (e.g., PICOS, funding sources) and any assumptions and simplifications made.	5
Risk of bias in individual studies	12	Describe methods used for assessing risk of bias of individual studies (including specification of whether this was done at the study or outcome level), and how this information is to be used in any data synthesis.	5
Summary measures	13	State the principal summary measures (e.g., risk ratio, difference in means).	5, 6
Synthesis of results	14	Describe the methods of handling data and combining results of studies, if done, including measures of consistency (e.g., I^2) for each meta-analysis.	5, 6
Risk of bias across studies	15	Specify any assessment of risk of bias that may affect the cumulative evidence (e.g., publication bias, selective reporting within studies).	5
Additional analyses	16	Describe methods of additional analyses (e.g., sensitivity or subgroup analyses, meta-regression), if done, indicating which were pre-specified.	5, 6
**RESULTS**			
Study selection	17	Give numbers of studies screened, assessed for eligibility, and included in the review, with reasons for exclusions at each stage, ideally with a flow diagram.	6, 7
Study characteristics	18	For each study, present characteristics for which data were extracted (e.g., study size, PICOS, follow-up period) and provide the citations.	7, 8 and supplement
Risk of bias within studies	19	Present data on risk of bias of each study and, if available, any outcome level assessment (see item 12).	8 and supplement
Results of individual studies	20	For all outcomes considered (benefits or harms), present, for each study: (a) simple summary data for each intervention group (b) effect estimates and confidence intervals, ideally with a forest plot.	9 to 12 and supplement
Synthesis of results	21	Present results of each meta-analysis done, including confidence intervals and measures of consistency.	10, 11, 12 and supplement
Risk of bias across studies	22	Present results of any assessment of risk of bias across studies (see Item 15).	8 and supplement
Additional analysis	23	Give results of additional analyses, if done (e.g., sensitivity or subgroup analyses, meta-regression [see Item 16]).	10, 12
**DISCUSSION**			
Summary of evidence	24	Summarize the main findings including the strength of evidence for each main outcome; consider their relevance to key groups (e.g., healthcare providers, users, and policy makers).	13
Limitations	25	Discuss limitations at study and outcome level (e.g., risk of bias), and at review-level (e.g., incomplete retrieval of identified research, reporting bias).	15

Conclusions	26	Provide a general interpretation of the results in the context of other evidence, and implications for future research.	15
**FUNDING**			
Funding	27	Describe sources of funding for the systematic review and other support (e.g., supply of data); role of funders for the systematic review.	16

From: Moher D, Liberati A, Tetzlaff J, Altman DG, The PRISMA Group (2009). Preferred Reporting Items for Systematic Reviews and Meta-Analyses: The PRISMA Statement. PLoS Med 6(7): e1000097. doi:10.1371/journal.pmed1000097

Cochrane risk of bias

Table S4 (a): Cochrane risk of bias of included Randomized Controlled studies

	Risk of bias domains					
Study	D1	D2	D3	D4	D5	Overall
Alexander et al. (2010)	+	-	-	+	+	-
Arija et al. (2017)	+	-	+	-	+	-
Ayala et al. (2015)	-	-	-	+	+	-
Baumann et al. (2015)	+	+	+	+	+	+
Bo et al. (2007)	+	-	+	+	+	-
Carrasquillo et al. (2017)	+	-	+	+	+	-
Chao et al. (2012)	+	-	-	-	+	-
Davies et al. (2016)	+	-	+	+	+	-
Elmer et al. (2006)	+	+	+	+	+	+
Havas et al. (2003)	+	+	+	+	+	+
Laska et al. (2016)	+	-	+	X	+	X
Lindström et al. (2003)	+	+	+	+	+	+
Lu CH et al. (2015)	+	-	+	+	+	-
Okube et al. (2022)	+	-	-	+	+	-
Østbye et al. (2009)	+	+	+	+	+	+
Ramachandran et al. (2013)	+	+	+	+	+	+
Takahashi et al. (2006)	+	-	+	+	+	-
van Keulen et al. (2021)	+	+	+	+	+	+
Wedick et al. (2015)	+	+	+	+	+	+
Woodruff et al. (2019)	+	-	+	+	-	-
Zhang et al. (2018)	+	-	+	+	+	-

Domains:
D1: Bias arising from the randomization process.
D2: Bias due to deviations from intended intervention.
D3: Bias due to missing outcome data.
D4: Bias in measurement of the outcome.
D5: Bias in selection of the reported result.

Judgement
X High
- Some concerns
+ Low

Figure S1 (a): Weighted summary of RoB for individual randomized controlled studies

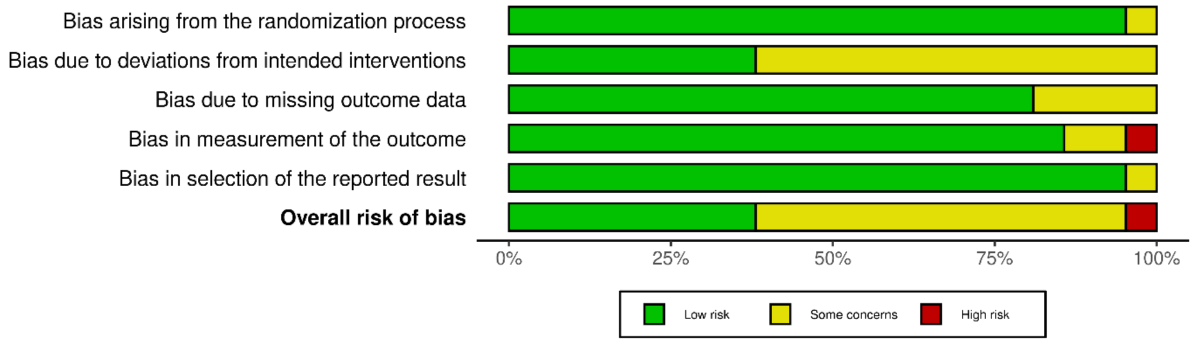

Table S4 (b): Cochrane risk of bias of included cluster randomized studies

Study	D1	D1b	D2	D3	D4	D5	Overall
Bóveda-Fontán et al. (2015)	+		-	+	+	+	-
Chandraratne et al. (2019)	+	+	-	+	+	+	-
Daivadanam et al. (2018)	+	+	-	+	-	+	-
Landry et al. (2015)	+		+	-	X	+	X
Dirige et al. (2013)	-	-	-	-	+	+	-
Fottrell et al. (2019)	+	-	+	+	-	+	-
Gunawardena et al. (2016)	+	+	+	+	-	+	-
Joshi et al. (2012)	+	-	X	+	-	+	X
Lombard et al. (2010)	+	+	-	+	+	+	-
Neupane et al. (2018)	+	+	+	-	+	+	-
Thankappan et al. (2018)	+	+	-	+	+	+	-
Yokokawa et al. (2020)	+	+	+	+	-	+	-

Risk of bias domains

Domains:
D1 : Bias arising from the randomization process.
D1b: Bias arising from the timing of identification and recruitment of Individual participants in relation to timing of randomization.
D2 : Bias due to deviations from intended intervention.
D3 : Bias due to missing outcome data.
D4 : Bias in measurement of the outcome.
D5 : Bias in selection of the reported result.

Judgement
X High
- Some concerns
+ Low
Not applicable

Table S4 (c): Cochrane risk of bias of included non-randomized studies

Study	D1	D2	D3	D4	D5	D6	D7	Overal
Anthony et al. (2015)	+	-	+	-	+	+	+	-
Ashfield-Watt et al. (2007)	+	-	+	+	+	+	+	-
Azizi et al. (2013)	+	+	+	+	-	-	+	-
Ibrahim et al. (2016)	+	-	+	+	-	+	+	-
Glasson et al. (2013)	+	+	+	+	+	+	+	+
Huang et al. (2011)	X	-	+	-	+	+	+	X
Kloek et al. (2006)	+	+	+	-	-	+	+	-
Koeder et al. (2022)	X	+	+	-	-	+	+	X
Luten et al. (2016)	-	X	+	-	+	+	+	-
Lv et al. (2014)	-	+	+	-	+	+	+	-
Mirmiran et al. (2008)	-	+	-	-	X	+	+	-
Nguyen et al. (2012)	-	-	-	-	-	+	+	-
Nishtar et al. (2007)	X	-	+	-	-	+	+	X
Ortega et al. (2016)	-	-	+	-	+	+	+	-
Sarrafzadegan et al. (2009)	+	-	+	+	+	+	+	-
Törmä et al. (2021)	+	+	+	+	+	+	+	+
Van de Vijver et al. (2016)	-	-	-	-	-	+	+	-
Wendel-Vos et al. (2009)	+	-	+	-	+	+	+	-

Risk of bias domains

Domains:
D1: Bias due to confounding.
D2: Bias due to selection of participants.
D3: Bias in classification of interventions.
D4: Bias due to deviations from intended interventions.
D5: Bias due to missing data.
D6: Bias in measurement of outcomes.
D7: Bias in selection of the reported result.

Judgement
X Serious
- Moderate
+ Low

Figure S1 (a): Weighted summary of RoB for non-randomized controlled studies

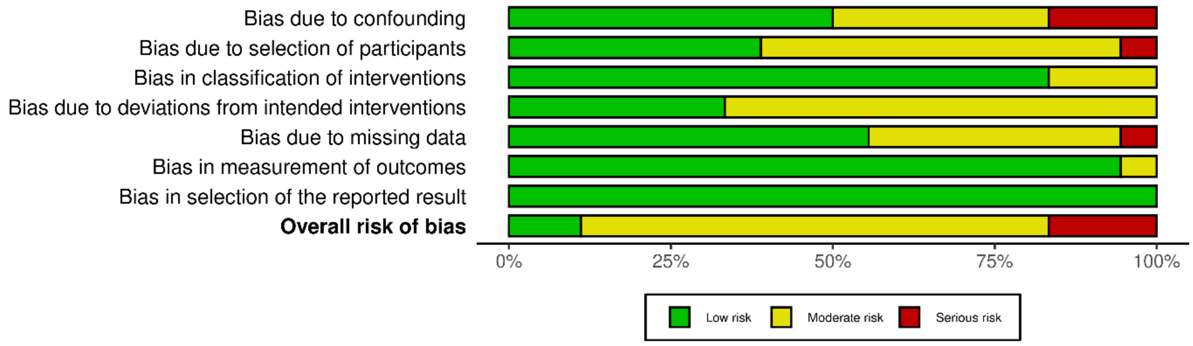

Subgroup analysis

By follow up time

Energy intake

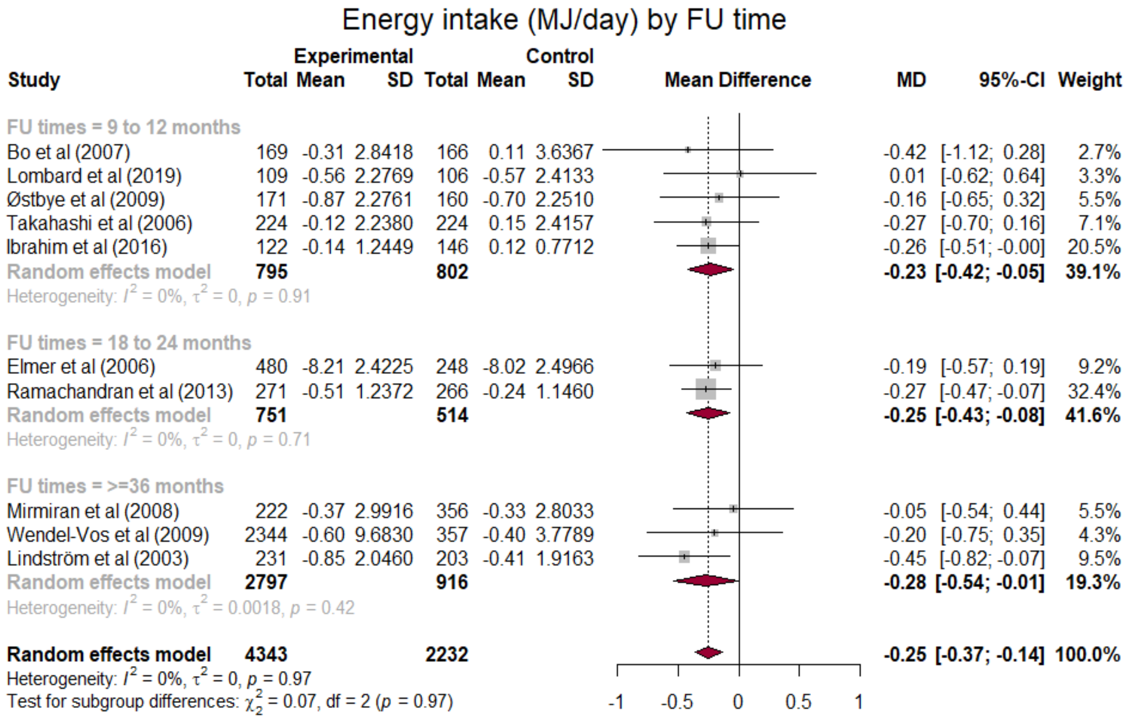

Figure S2. Subgroup analysis of energy intake (MJ/day) over FU time

Fiber intake

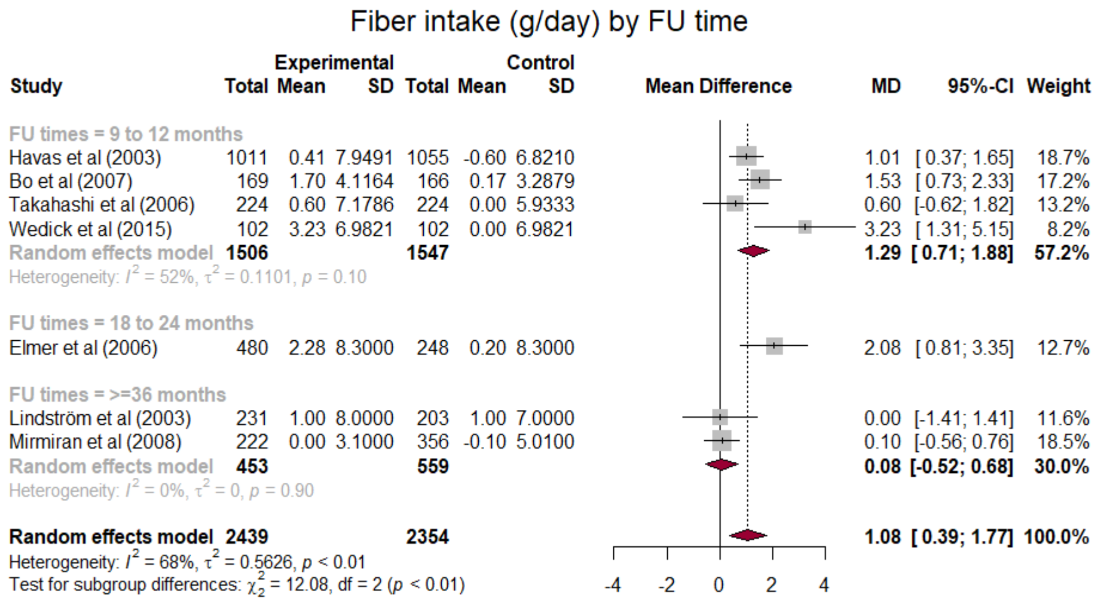

Figure S3. Subgroup analysis of fiber intake (MJ/day) over FU time

Fruit and vegetable intake

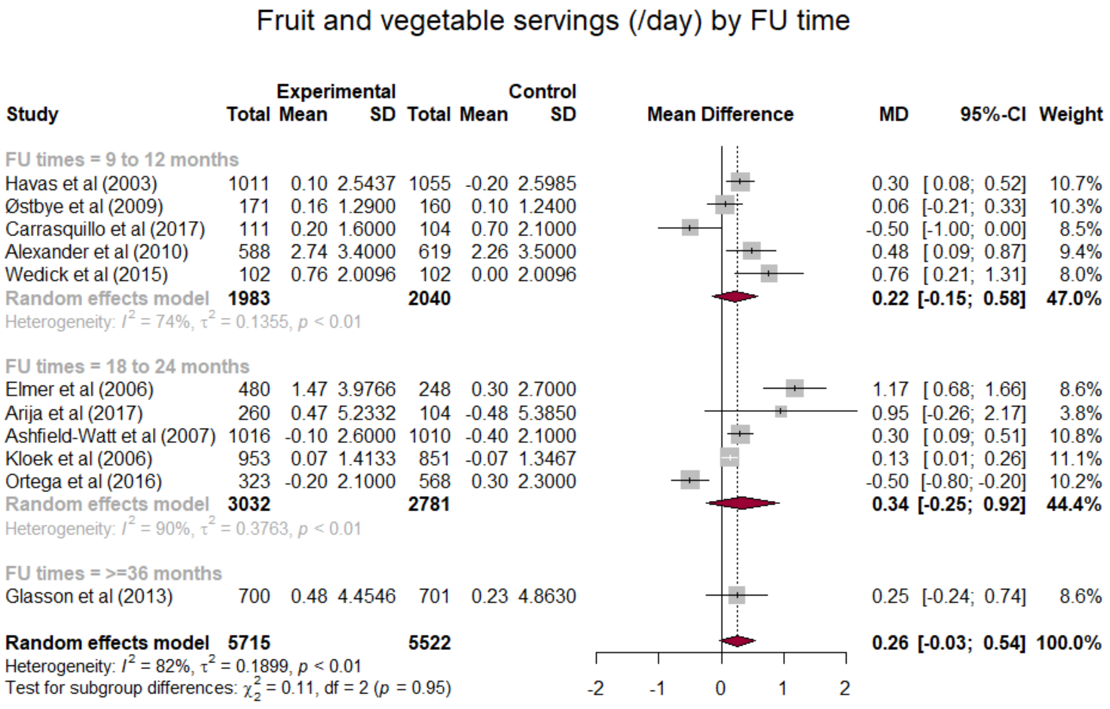

Figure S4. Subgroup analysis of fruit and vegetable intake (MJ/day) over FU time

Fat % of energy

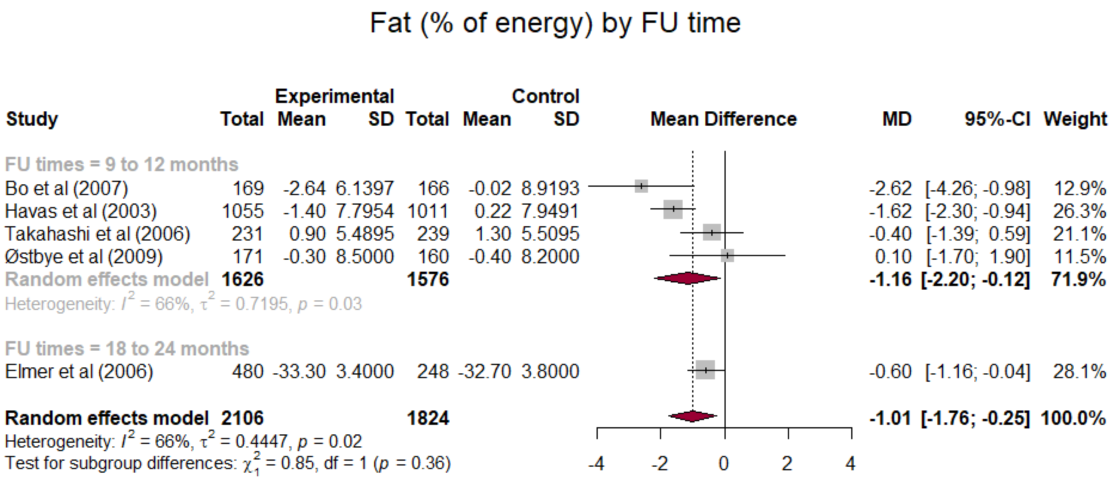

Figure S5. Subgroup analysis of fat intake (% of energy) over FU time

By study design

Energy intake

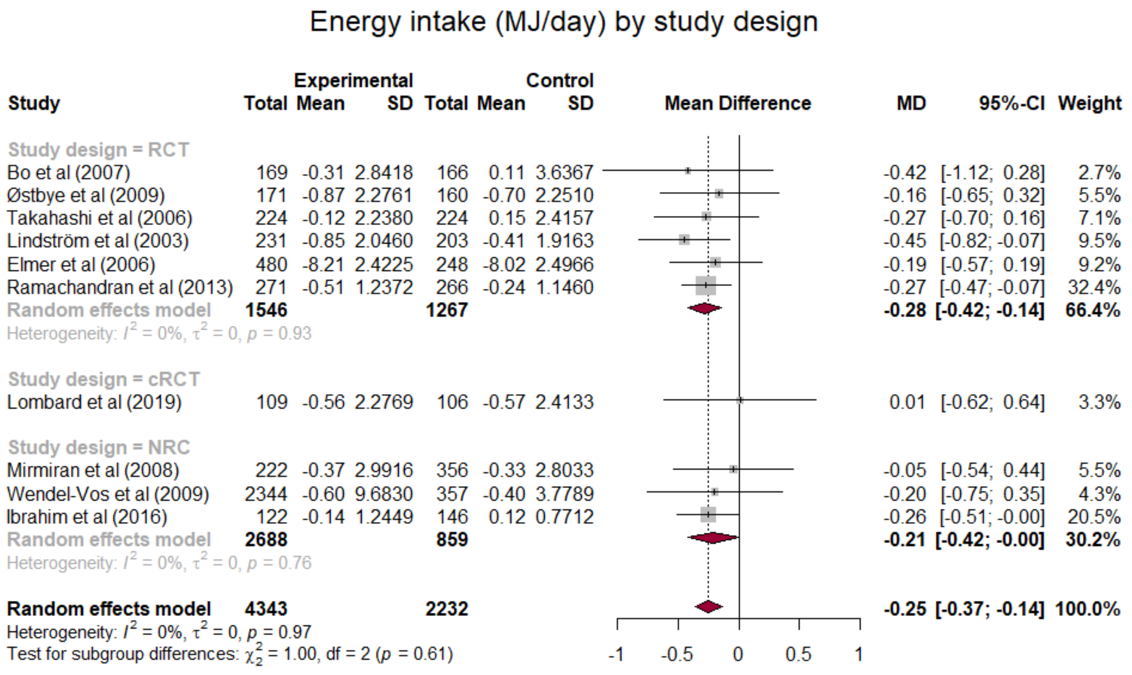

Figure S6. Subgroup analysis of energy intake (MJ/day) by study design

Fiber intake

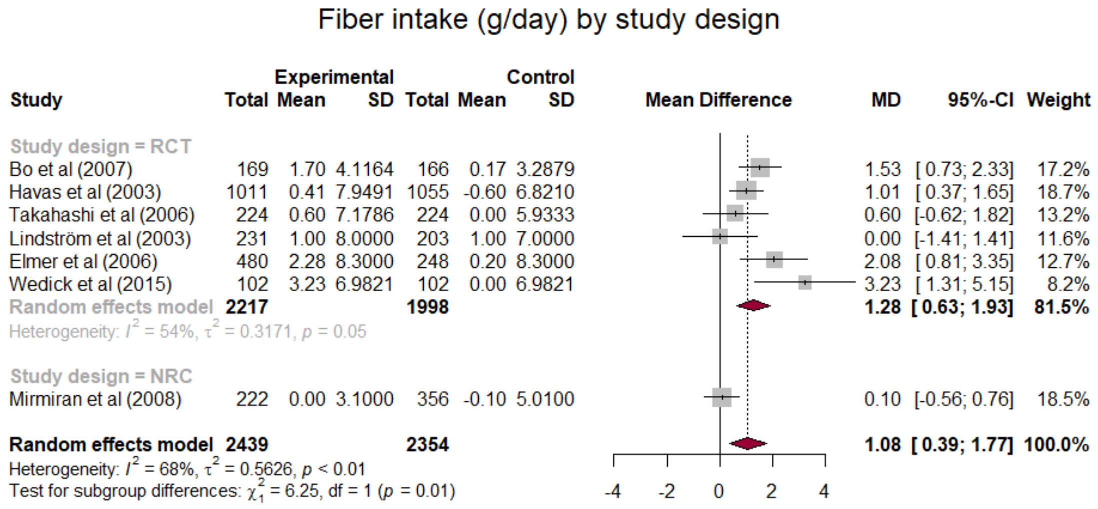

Figure S7. Subgroup analysis of fiber intake (g/day) by study design

Fruit and vegetable intake

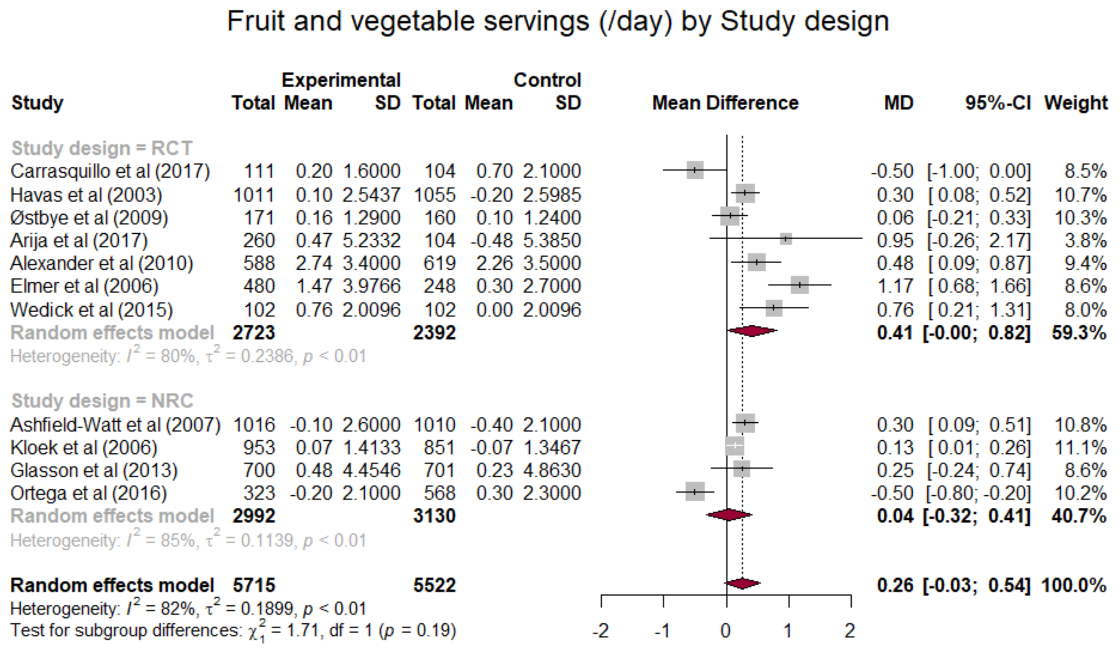

Figure S8. Subgroup analysis of fruit and vegetable servings (/day) by study design

Publication bias assessment

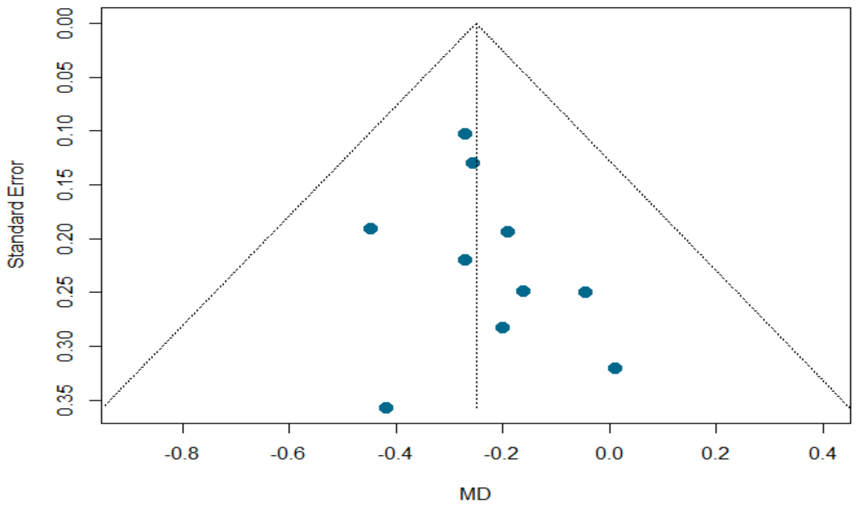

a)

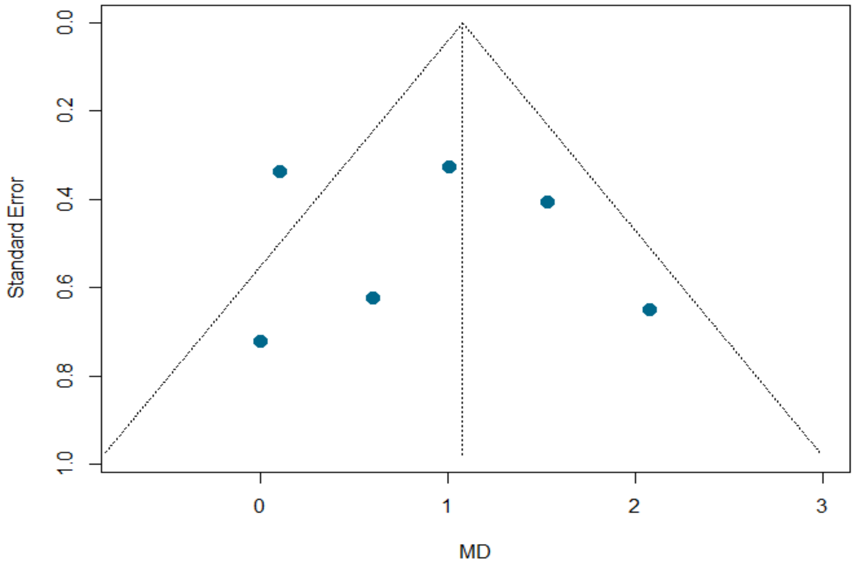

b)

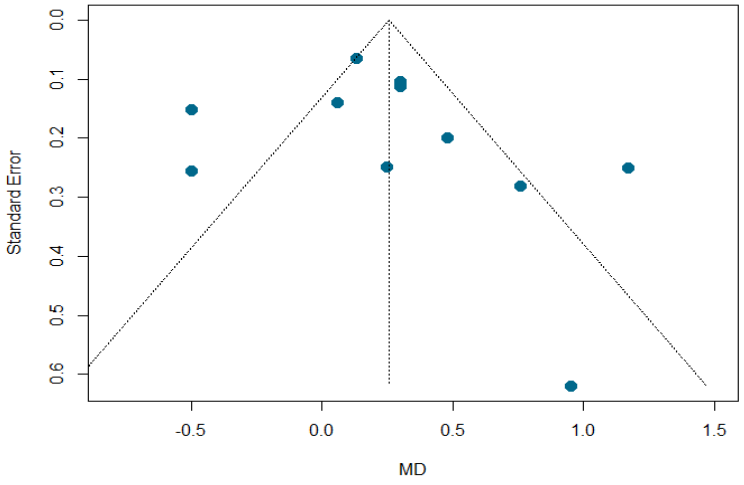

c)

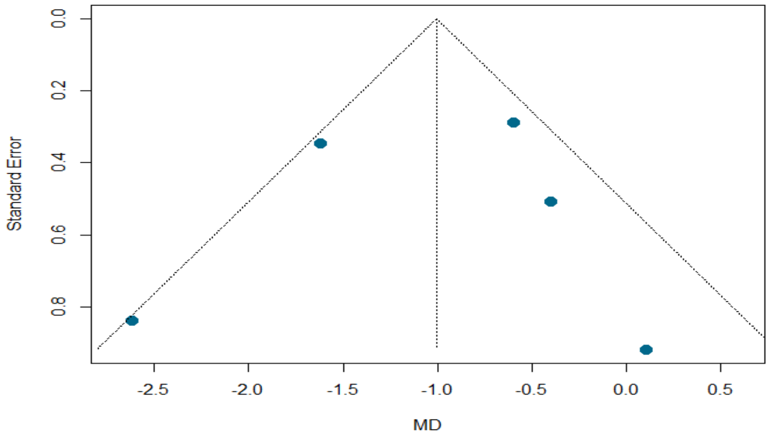

d)

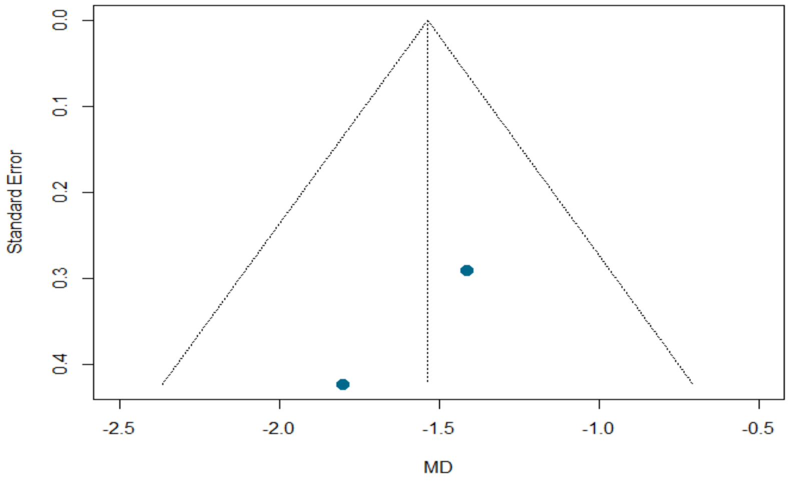

e)

Figure S7. Funnel plots of energy intake (p = 0.392), b) fiber intake (p = 0.332), c) fruit and vegetable intake (p = 0.485), d) fat % of energy (p = 0.855), and e) saturated fat % of energy (p = NA)

Search strategy

Box S1: Search strategy in Medline

#1	("Community" OR "community-based intervention" OR "community-based" OR "community based" OR "community intervention" OR "population-based intervention" OR "population based" OR "population intervention" OR "community health" OR "community organisation" OR "community organization" OR "community program*" OR "Community level" OR "Community networks" OR "community health services" OR "home based" OR "community participation" OR "community-based research")
#2	(Interven* OR strateg* OR approach* OR program* OR "health education" OR "health educ*" OR advise OR "raising awareness" OR counsel* OR "health promotion" OR "health campaign" OR "wellness program*" OR "mass media" OR "behaviour* change" OR "behavior* change" OR "lifestyle intervention" OR "lifestyle program*" OR "screening" "motivational interviewing" OR "risk scoring" OR refer* OR training OR "capacity building" OR "peer" OR "peer group" OR "community health worker" OR "CHW" OR "community health volunteer" OR "health worker*" OR "Community Health Extension Worker" OR "Health promoter" OR "Community Health Care Provider" OR "social support" OR "adherence support" OR "coaching" OR "self management" OR self-management OR "outreach" OR "home visit" OR "appointment reminders")
#3	("Cardiovascular disease" OR "CVD" OR "CVD risk" OR "cardiovascular disease prevention" OR "cardiovascular disease control" OR "stroke" OR "coronary heart disease" OR "heart diseas*" OR "heart failure" OR "kidney disease" OR "Cardiovascular risk factor" OR "hypertension" OR "raised blood pressure" OR diabetes OR "raised blood sugar" OR "cholest*" OR triglyceride OR HDL OR LDL OR "lipid profile" OR "metabolic syndrome" OR "body mass index" OR "BMI" OR "Overweight" OR "obesity" OR "obese" OR "waist circumference" OR "life style" OR "lifestyle" OR "alcohol" OR "tobacco" OR "smoking" OR "diet*" OR "nutrition" OR "food habit" OR "junk food" OR "fast food" OR "fruit" OR "vegetables" OR "five a day" OR "salt reduction" OR "physical inactivity" OR "physical activity" OR "exercise" OR "stress")
#4	("randomized controlled trial" OR "randomized" OR "randomised" OR "controlled study" OR trial OR RCT OR cluster OR CRT OR "comparative study" OR "quasi experimental study" OR "quasi-experiment" OR "experimental" OR "control group" OR "follow up" OR "prospective" or "retrospective" OR placebo OR random* OR "follow-up" OR "non-random*" OR "nonrandom*" OR "before after stud*" OR "before and after" or "time series" or "time-series" OR "interrupted time series" OR longitud* OR "controlled before" OR "pre-post" OR pretest OR posttest OR "pre intervention" or "post intervention")
#4	#1 AND #2 AND #3 AND #4
#5	<u>Filters:</u> **year of publication:** (January 2000 to June 2022), **Language:** English **Age:** adults (18 and above) **population:** humans
